# Comment on: Martelletti et al. Refractory chronic migraine: a consensus statement on clinical definition from the European Headache Federation

**DOI:** 10.1186/1129-2377-15-77

**Published:** 2014-11-24

**Authors:** Christian Wöber, Peter Wessely

**Affiliations:** 1 Department of Neurology, Medical University of Vienna, Währinger Gürtel 18-20, 1090 Vienna, Austria; 2 Neurological Office, Vienna, Austria

## Abstract

In this letter, we present the Austrian proposal for diagnostic criteria of refractory chronic migraine and we discuss the consensensus statement of the European Headache Feaderation. We focus in particular on the definition of adequate prophylactic treatment, the management of medication overuse and the requirement for CSF analyses in patients with refractory chronic migraine. In our proposal, the criteria for adequate treatment and recommendations for dealing with medication overuse are more explicit than in the EHF proposal, whereas the requirements for CSF analyses and measurement of CSF pressure are not as strict.

## Correspondence/Findings

We read with great interest the consensus statement on refractory chronic migraine (rCM) from the European Headache Federation (EHF) [1]. In parallel to EHF, a group of neurologists experienced in the management of chronic migraine (CM) as well as neurosurgeons and anaesthesiologists with expertise in neuromodulation prepared a consensus statement on rCM and patient selection for occipital nerve stimulation (ONS) for Austria [2]. We focused on ONS, since it is currently the only neuromodulation technique for rCM examined in randomized controlled studies [3-5].

In this comment we want to present the Austrian proposal for diagnostic criteria of rCM, suggest revisions of the “notes” in the EHF statement, summarize our recommendations for selecting ONS candidates and discuss critical issues of rCM diagnostic criteria.

### Diagnostic criteria

(A) CM according to ICHD-3-beta for at least 24 months causing significant impairment of quality of life and/or socio-economic burden. (B) Modification of trigger factors and lifestyle and treatment of comorbid disorders did not improve the headache. (C) At least 3 adequate treatments with prophylactic medication were unsuccessful. (1) “Unsuccessful“ is defined by (a) no or insufficient efficacy (based on patient report and recordings in a headache diary), (b) intolerable adverse effects, (c) contraindications. (2) Adequate treatment requires the intake of specific compounds (a) from certain classes of drugs, (b) in an effective dosage, (c) over a period of at least free months. (3) Classes of drugs (a) beta blockers: propranolol 80-160 mg, metoprolol 100-200 mg, bisoprolol 5-10 mg, (b) anticonvulsants: topiramate 75-100 mg, valproic acid 600-1500 mg (c) tricyclics: amitriptyline up to 75 mg, (d) flunarizine: 5–10 mg, (e) other drugs with at least one positive randomised controlled study - e.g. lisinopril 20 mg, candesartan 20 mg, onabotulinumtoxin A 155 – 195U according to PREEMPT. (4) Use of drugs from at least three of the classes a-d [6,7].

### Notes

(1) In patients with medication overuse (MO) according to ICHD-3 beta, outpatient or inpatient detoxification and diagnostic re-evaluation after two months of follow-up is mandatory. (2) Other primary headaches such as hemicrania continua and new daily persistent headache as well as secondary headaches apart from medication overuse headache must be excluded by history, clinical examination and laboratory analyses. (3) Cranial MRI including the cranio-cervical region, MR-angiography and MR-venography do not show a disorder explaining the headache. (4) CSF pressure should be measured in patients with evidence of sinus stenosis in MR venography [1].

### Recommendations

Patient selection and management before ONS should comprise confirmation of refractory CM and exclusion of ONS contraindications, detoxification in case MO, patient information about ONS, exclusion of disorders explaining the headache or preventing ONS, and detailed written documentation (modified according to [8]).

### Discussion

In rCM, there are two crucial points. First, adequate prophylactic treatment of migraine and second, co-existing MO. With respect to pharmaco-prophylaxis we tried to be as specific as possible. In contrast to the EHF criteria [1] requiring “at least 3 different drugs from the following classes” and thus allowing diagnosis of rCM for example after non-response to three different betablockers, we think that the use of drugs from different classes should be mandatory [2,6]. Furthermore, we specified the required minimum dose for almost all prophylactic drugs and did not just give a maximum dose as EHF did.

Regarding MO in CM there are four possible scenarios, (1) no previous or current MO, (2) previous but no current MO, (3) current without previous MO and (4) previous as well as current MO. We believe that none of these scenarios should *a priori* prevent the diagnosis of rCM or subsequently the selection for invasive neuromodulation. In parallel to the ICHD-3 beta criteria of CM, patients with rCM and MO could initially have both diagnoses before the response to detoxification allows confirming or rescinding the diagnosis of MO [9].

In our opinion, the EHF proposal is inconsistent with respect to MO, since criterion A requires no MO, but recommendations for detoxification are given in the “notes”. In addition, we believe that a follow-up period after detoxification should be specified. We also want to comment on CSF analysis and measurement of CSF pressure. The “notes” given by EHF suggest that CSF analyses are mandatory in all patients with rCM. We think, this is not justified. The same is true for the measurement of CSF pressure. The possible importance of intracranial hypertension in patients with “unresponsive chronic migraine” has only been suggested in one single case series of 44 patients [10]. Further studies are necessary to confirm these findings. For the time being, measurement of CSF pressure may be performed selectively in CM patients with abnormal findings in MR venography, but not routinely in all patients with CM.

### Conclusion

Diagnostic criteria for rCM and guidelines for managing patients with rCM and selecting candidates for invasive neuromodulation are crucial issues. This comment may contribute to the ongoing discussion promoted substantially by the EHF consensus statement.

## Competing interests

For the first meeting, all members of the consensus team received a honorarium from St. Jude Medical Medizintechnik Austria. The consensus statement and its entire content was prepared exclusively by the consensus team.

## Authors’ contributions

Both authors read and approved the final manuscript.
